# Synthesis and Migration-Plugging Performance of a Novel High-Temperature Resistant Epoxy-Based Profile Control Agent for Heavy Oil Steam Flooding

**DOI:** 10.3390/polym18172133

**Published:** 2026-09-01

**Authors:** Jinxiang Liu, Xianpei Yin, Yifei Gao, Xiangguo Lu, Hongwen Zhang, Hongyu Wang, Qiuxia Wang, Hao Liu

**Affiliations:** 1 Key Laboratory of Enhanced Oil and Gas Recovery of Ministry of Education, Northeast Petroleum University, Daqing 163318, China; liujinxiangdbsy@163.com (J.L.); yinxianpei620@163.com (X.Y.); gyf20000224@163.com (Y.G.); 2 CNOOC Key Laboratory of Offshore Heavy Oil Thermal Recovery, Tianjin 300452, China; zhanghongwendbsy@163.com (H.Z.); wanghongyudbsy@163.com (H.W.); wangqiuxiadbsy@163.com (Q.W.); liuhaodbsy@163.com (H.L.)

**Keywords:** epoxy composite, imide-triazine modification, nano-silica reinforcement, liquid microspheres, high-temperature resistance, deep conformance control, heavy oil steam flooding, enhanced oil recovery

## Abstract

Severe steam channeling in heterogeneous heavy oil reservoirs severely restricts thermal recovery efficiency, as conventional conformance control materials cannot simultaneously achieve long-term high-temperature resistance and deep reservoir penetration. This work develops a high-temperature-resistant epoxy-based liquid microsphere system for deep profile control in heavy oil steam flooding. A triple thermal-stabilization strategy is constructed: imide chain extension to enhance backbone rigidity, benzoxazine-phthalonitrile grafting to form dense triazine crosslinked networks, and KH-550-functionalized nano-silica for synergistic reinforcement. FT-IR and ^1^H NMR verify the successful incorporation of rigid imide and triazine structures. TGA confirms the optimal formulation exhibits less than 5% mass loss at 350 °C. Multi-segment sand-packed tube tests demonstrate favorable deep migration capacity with inter-stage pressure ratios below 4 across 5000–15,000 × 10^−3^ μm^2^ permeability, and the cured network retains over 95% plugging efficiency after 350 °C steam scouring. Dual-tube heterogeneous flooding delivers 12.05% incremental oil recovery, outperforming rigid inorganic particles. This system provides a high-performance candidate for deep steam channeling mitigation in heavy oil thermal recovery.

## 1. Introduction

Heavy oil constitutes an essential strategic energy resource in China, with massive proven reserves and large-scale thermal recovery operations relying primarily on cyclic steam stimulation and continuous steam flooding. Continuous high-temperature steam injection effectively reduces crude viscosity and supplements reservoir energy, yet severe interwell steam channeling universally arises in heterogeneous heavy oil formations characterized by loose sandstone matrices and high oil viscosity. Steam channeling drastically reduces steam sweep efficiency and thermal utilization, severely restricting the economic performance of steam flooding. Accordingly, developing high-performance conformance control materials to mitigate steam breakthrough and enlarge swept volumes has become an urgent research target for thermal heavy oil exploitation [1].

Three mainstream classes of profile control agents, including polymer gels, rigid inorganic particles, and foams, have been widely investigated for steam channeling remediation [2]. Polyacrylamide-based organic gels exhibit low cost and minor formation damage, yet they suffer irreversible thermal decomposition and structural collapse under prolonged high-temperature steam exposure, failing to sustain long-term plugging above 200 °C [3,4]. Inorganic rigid fillers such as quartz sand possess outstanding thermal and erosion resistance, but their non-deformable solid morphology readily induces near-wellbore bridging retention, which prevents deep penetration into reservoir porous media and leads to subsequent steam bypass. Foam agents integrate both shutoff and oil-washing functions, while their fragile bubble frameworks rapidly degrade under high-temperature conditions, limiting large-scale field deployment [5]. Collectively, conventional conformance materials cannot concurrently satisfy the dual demands of high-temperature steam tolerance and in-depth reservoir migration, creating a critical technical bottleneck for thermal recovery [6].

Epoxy polymers exhibit outstanding adhesion and customizable molecular architectures, and crosslinked epoxy solids can deform and propagate through pore channels, making them promising candidates for deep conformance control [7]. Nevertheless, commercial bisphenol A epoxy resins display insufficient thermal stability and degrade rapidly at temperatures exceeding 300 °C, restricting their application in high-temperature steam flooding formations. Molecular structural modification combined with nanofiller synergy represents a feasible strategy to elevate the thermal and mechanical performances of epoxy matrices. Incorporating benzoxazine-phthalonitrile moieties generates dense triazine crosslinking networks upon curing; imide rigid segments strengthen polymer backbone rigidity; and surface-functionalized nano-silica establishes tortuous thermal diffusion pathways within the composite [8,9]. The synergistic combination of three thermal-stabilization pathways is anticipated to fabricate advanced epoxy composites integrating superior heat resistance and deep migration capability [10,11,12,13,14,15].

Herein, a novel high-temperature-resistant epoxy-based liquid microsphere conformance system was synthesized using bisphenol A epoxy as the primary matrix. The composite was modified via imide chain extension, grafted with benzoxazine-containing phthalonitrile monomers, and blended with KH-550-functionalized nano-silica. Fourier transform infrared spectroscopy (FT-IR), proton nuclear magnetic resonance (^1^H NMR), and thermogravimetric analysis (TGA) were utilized to characterize molecular structures and optimize composite formulations. Multi-section pressure-monitored sand-pack tubes and dual parallel heterogeneous core flooding setups were adopted to systematically assess the in-depth migration, high-temperature steam erosion resistance, and oil-displacement performance of the prepared epoxy microspheres, and the underlying working mechanisms were elucidated. This work offers a novel polymer composite candidate and reliable experimental support for deep steam channeling mitigation in heavy oil steam flooding reservoirs [16,17,18,19,20].

## 2. Experimental Section

### 2.1. Instruments and Materials

The following reagents were used in the experiments: 2,2-bis(4-hydroxyphenyl) propane (bisphenol A, BPA), epichlorohydrin, γ-aminopropyltriethoxysilane (KH-550), nano-silica (particle size: 15–30 nm), N, N-dimethylformamide (DMF), N-methylpyrrolidone (NMP), benzene, sodium hydroxide, silver nitrate, pyromellitic dianhydride (PMDA), 2,2-bis(3-amino-4-hydroxyphenyl) propane, and triphenylphosphine (TPP). All commercial reagents were of analytical reagent grade and purchased from Sinopharm Chemical Reagent Co., Ltd., Shanghai, China. The oxazine-containing phthalonitrile monomer (BPS-PH) was self-synthesized in the laboratory. Partially hydrolyzed polyacrylamide (HPAM, molecular weight: 1.9 × 10^7^, effective content: 90%) was obtained from Daqing Refining & Chemical Company, Daqing, China. The 3000 mg/L HPAM solution was prepared using simulated injection water as the solvent, with an initial viscosity of 762 mPa·s at 65 °C. After high-speed shearing at 12,000 r/min for 10 s, its viscosity retention rate generally ranged from 53% to 58% under conventional test conditions. Refined quartz sand was adopted as the inorganic particle control sample, supplied by Lingshou Anda Mineral Powder Factory (Shijiazhuang, China). It has a median particle size of 30 μm and a density of 2.65 g/cm^3^, with high-temperature resistance meeting the requirements of 350 °C steam working conditions. Simulated injection water was used as the aqueous phase, with a total salinity of 2152 mg/L. Its detailed ionic composition is presented in Table 1. The experimental simulated oil was prepared by blending dehydrated crude oil from Bohai N Oilfield, Tianjin, China with kerosene, with a measured viscosity of 500 mPa·s at 65 °C.

The main testing and displacement apparatus included a Fourier transform infrared spectrometer (Thermo Fisher Scientific, Shanghai, China), a DSC3500 thermogravimetric analyzer (NETZSCH, Selb, Germany), a 500 MHz nuclear magnetic resonance spectrometer (Bruker, Karlsruhe, Germany), a Quanta450 field emission environmental scanning electron microscope (FEI, Hillsboro, OR, USA), a Brookfield LVDV-II rotational viscometer (Brookfield, Middleboro, MA, USA), and a Waring high-shear blender (Waring, Stamford, CT, USA).

**Figure 1:**
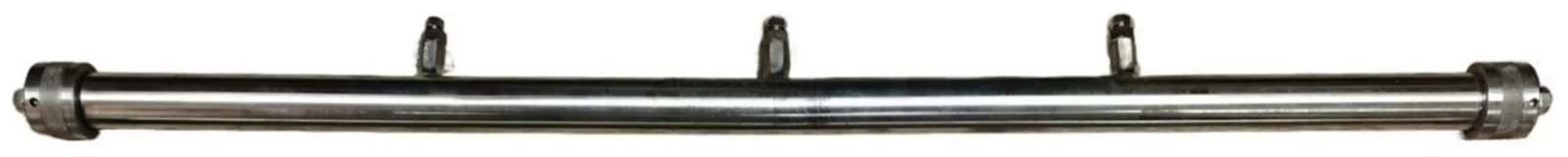
Photos of sand-packed tubes for migration and transport experiments.

**Figure 2:**
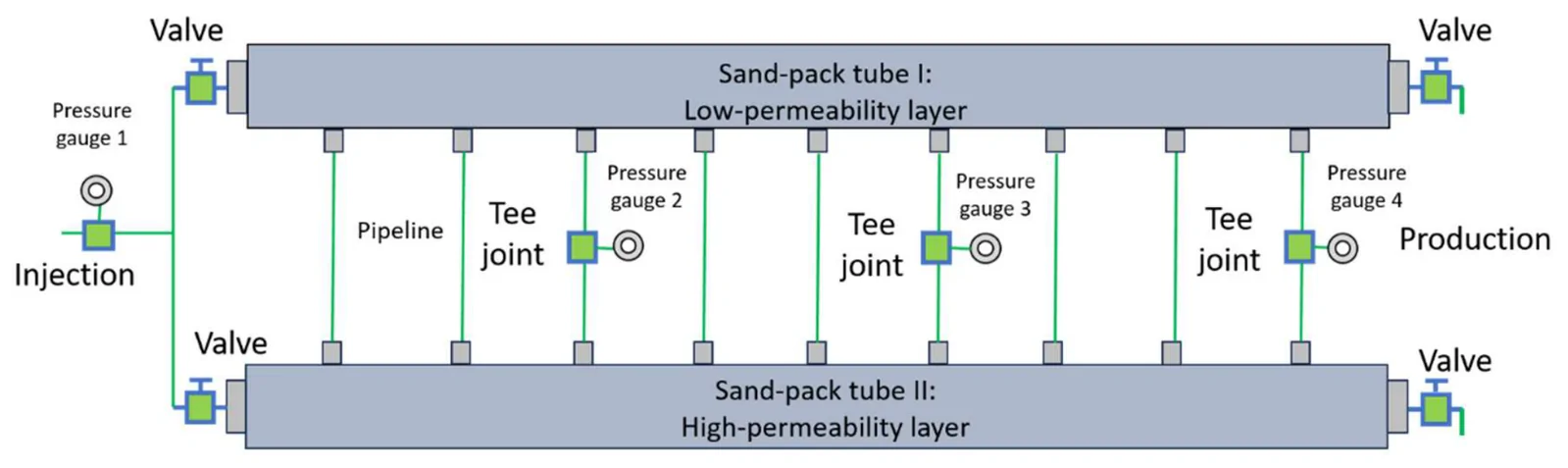
Schematic diagram of dual parallel tube model apparatus.

A self-fabricated multi-pressure-point sand-packed tube (3 cm in diameter, 100 cm in length) was adopted, equipped with 4 pressure monitoring points along the flow path, located at the injection end, 0.25 m, 0.5 m and 0.75 m from the inlet, respectively. The photograph of the sand-packed tube is shown in Figure 1. Other supporting equipment included a constant-flux pump, pressure transducers, intermediate vessels, a steam generator and a thermostatic chamber. All components of the displacement system except the constant-flux pump were placed in the thermostatic chamber maintained at 65 °C, with a temperature control accuracy of ±0.5 °C. The schematic diagram of the dual-tube parallel model setup is shown in Figure 2.

### 2.2. Preparation of the High-Temperature Resistant Epoxy-Based Profile Control and Flooding System

Using bisphenol A epoxy resin as the matrix, the high-temperature resistant profile control system was prepared through three modification steps: imide chain extension, oxazine ring crosslinking, and nanofiller reinforcement. The detailed procedures are as follows.

#### 2.2.1. Synthesis of Base Epoxy Prepolymer and Chain Extension Modification

The bisphenol A epoxy prepolymer was synthesized via a two-step method. Bisphenol A and epichlorohydrin were charged into a 250 mL four-necked flask at a molar ratio of 1:3, with the mechanical stirring speed set at 250 rpm. The system was heated to 65–70 °C in a water bath. After the solids were completely dissolved, a 30 wt% NaOH solution was slowly added dropwise over 0.5 h (the total molar amount of NaOH was 2.1 times that of bisphenol A, with 70% of the total amount added in the first batch). The system temperature was controlled at 70–75 °C to avoid hydrolysis of epichlorohydrin. After the dropwise addition, the temperature was raised to 75–80 °C and maintained for 1.5–2 h to complete the ring-opening etherification reaction, yielding chlorohydrin ether intermediates.

Subsequently, the reaction system was cooled to room temperature, and the remaining 30% NaOH solution was slowly added dropwise. Equal volumes of benzene and distilled water were added for extraction and phase separation. The organic phase was washed repeatedly with distilled water until the aqueous phase was neutral in pH and free of Cl^−^ residue, as verified by AgNO_3_ solution. The washed organic phase was transferred to a rotary evaporator. Benzene and unreacted epichlorohydrin were first distilled off at atmospheric pressure, followed by vacuum distillation at an absolute pressure of 5 mmHg and 110–120 °C for 2 h to remove residual volatiles. The product was poured out while hot and cooled naturally to room temperature, obtaining an amber, transparent, and viscous bisphenol A epoxy prepolymer.

To further improve the thermal stability by introducing rigid imide segments into the polymer backbone, equimolar amounts of pyromellitic dianhydride (PMDA) and 2,2-bis(3-amino-4-hydroxyphenyl) propane were dissolved in NMP solvent with a solid content of 25 wt%. Under continuous nitrogen protection (flow rate: 15 mL/min) and mechanical stirring at 250 rpm, the ring-opening addition reaction was carried out at room temperature for 2 h. During this stage, the aromatic primary amine groups on the diamine reacted with anhydride moieties to form amide bonds and adjacent carboxyl groups, affording a polyamic acid (PAA) prepolymer intermediate.

Subsequently, the system was heated to 160–180 °C and held for 2 h to perform thermal dehydration cyclization. The ortho-positioned amide and carboxyl groups underwent intramolecular cyclization with the elimination of two molecules of water, forming two five-membered imide rings. The nitrogen atoms derived from the initial amine groups were fully retained within the cyclic imide backbone, while the phenolic hydroxyl groups on the benzene rings remained unreacted for the subsequent chain-extension reaction with epoxy prepolymer. After the reaction, the system was cooled to room temperature and slowly poured into 10 volumes of deionized water for precipitation. After suction filtration, the filter cake was washed three times with anhydrous ethanol (10 min per wash) and dried in a vacuum drying oven at 80 °C under a gauge pressure of −0.1 MPa for 12 h, yielding the rigid imide-containing aromatic diphenol heat-resistant monomer (IA).

The heat-resistant monomer and base epoxy prepolymer were weighed at a molar ratio of hydroxyl groups to epoxy groups of 1:1 and added to the reactor, along with 0.5–1 wt% triphenylphosphine relative to the total mass of the resin as a catalyst. The reaction was carried out at 160–170 °C and 250 rpm for 3 h under nitrogen protection. The ring-opening addition reaction between phenolic hydroxyl groups and epoxy groups completed the chain extension modification, obtaining chain-extended epoxy resin with imide structures in the backbone.

**Figure 3:**
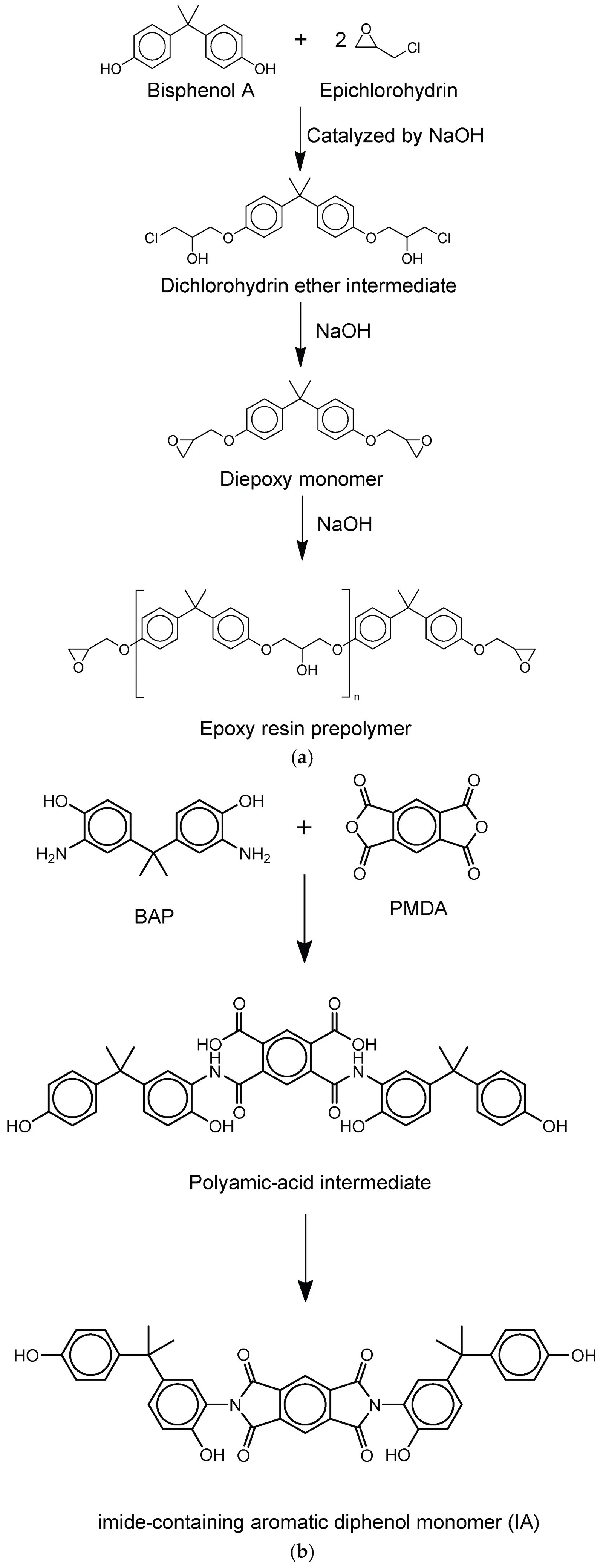

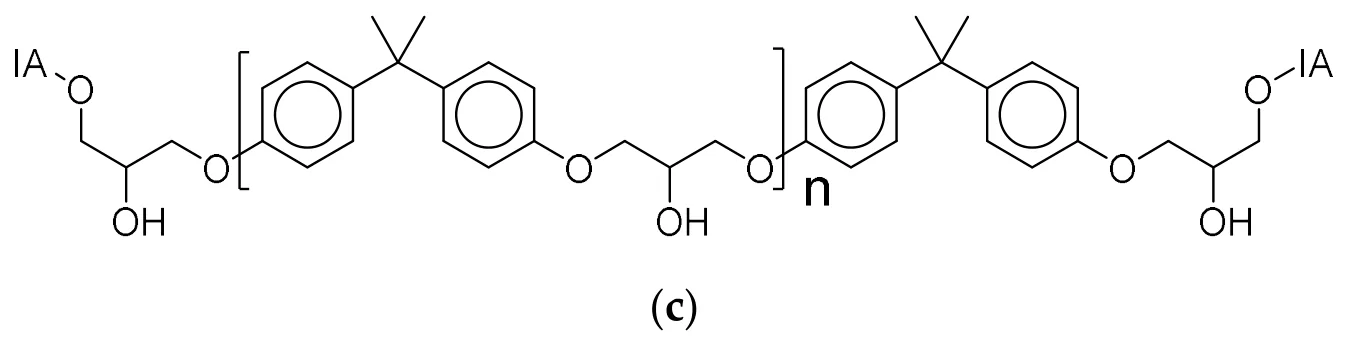
Synthetic routes and corresponding molecular structures of imide chain-extended epoxy resin. (**a**) Synthesis of epoxy resin prepolymer via ring-opening etherification and ring-closing reactions; (**b**) two-step synthetic route of imide containing aromatic diphenol monomer (IA). The polygamic-acid intermediate was formed via room-temperature ring-opening addition, and the final cyclized imide product (IA) was obtained through thermal dehydration cyclization; (**c**) chain-extension product of epoxy prepolymer modified with imide-containing aromatic diphenol monomer (IA).

The complete synthetic route and corresponding molecular structures of the above chain extension modification process are schematically presented in Figure 3.

#### 2.2.2. Nanofiller Pretreatment and Synthesis of BPS-PH Monomer

Surface amino modification of nano-SiO_2_ was performed using KH-550 (Sinopharm, Shanghai, China) as the coupling agent. Nano-SiO_2_ was dispersed in anhydrous ethanol to prepare a suspension with a mass fraction of 5%, which was placed in an ultrasonic cleaner and subjected to ultrasonic treatment at 300 W and 40 kHz for 30 min to form a uniform dispersion. KH-550 was added at a dosage of 10% of the nano-SiO_2_ mass, and the reaction was conducted at 70 °C with stirring at 250 rpm for 2–4 h. After the reaction, the suspension was centrifuged at 8000 rpm for 10 min. The supernatant was discarded, and the precipitate was washed three times with anhydrous ethanol to remove the unreacted coupling agent. The collected product was dried in a vacuum drying oven at 80 °C and an absolute pressure of −0.1 MPa for 12 h. After grinding, a surface amino-modified nano-SiO_2_ powder was obtained and stored in a sealed container for later use.

The oxazine-containing phthalonitrile (BPS-PH) monomer was synthesized via the solvent method. Bisphenol S, 3-aminophthalonitrile and paraformaldehyde (calculated as formaldehyde) were fed at a molar ratio of 1:2:4 into toluene solvent, with the solid content controlled at 20 wt%. The four-necked flask was equipped with a Dean-Stark trap (BOMEX, Beijing, China), a condenser and a nitrogen inlet. Under continuous nitrogen protection and stirring at 250 rpm, the system was heated to 110–115 °C for a reflux azeotropic reaction for 4–6 h until no more water was generated in the Dean-Stark trap. After the reaction, the system was cooled to 60 °C, and the reaction solution was slowly poured into 8 volumes of cold isopropanol for precipitation. After suction filtration, the product was washed twice with cold isopropanol and dried in a vacuum drying oven at 50 °C and an absolute pressure of −0.1 MPa for 12 h, yielding light yellow BPS-PH monomer powder.

**Figure 4:**
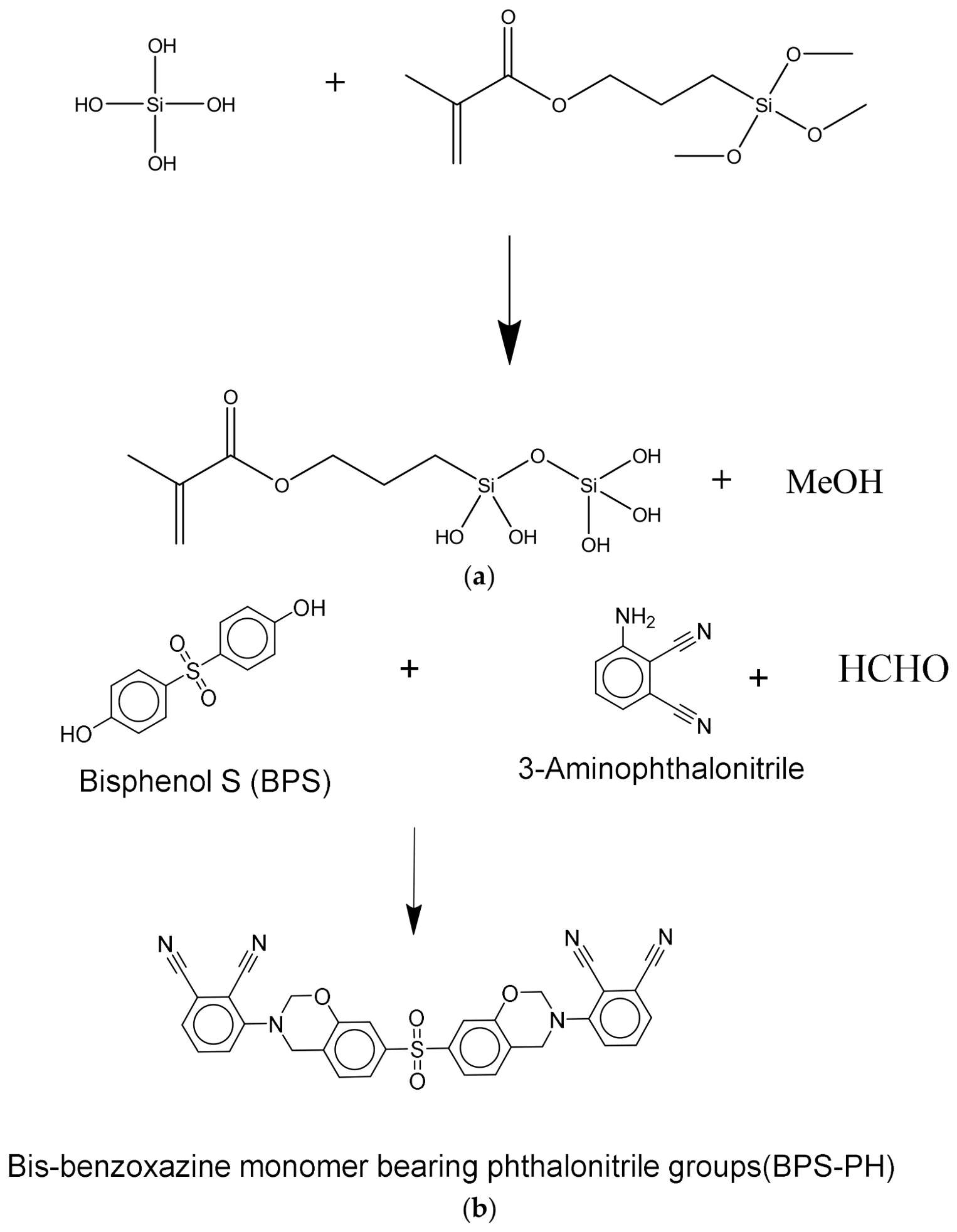
Preparation schematics of modified nano-silica and BPS-PH functional monomer. (**a**) Surface modification of nano-silica; (**b**) synthesis of BPS-PH prepolymer.

The modification procedure of nano-SiO_2_ and the synthetic route of the BPS-PH monomer are schematically described in Figure 4.

#### 2.2.3. System Formulation

Based on the mass of the chain-extended epoxy resin, the BPS-PH monomer was weighed according to the preset ratio and dissolved in an appropriate amount of NMP, with the solid content controlled at approximately 50 wt%. After stirring at 60–80 °C and 200 rpm until the monomer was completely dissolved, the chain-extended epoxy resin was added, and stirring was continued for 30 min until the system was homogeneous. The modified nano-SiO_2_ powder was slowly added, followed by 15 min of mechanical stirring and 20 min of ultrasonic dispersion at 300 W to ensure no filler agglomeration.

The system was cooled to room temperature, and curing agent KH-550 (dosage calculated relative to the total resin mass) was added, followed by stirring at 200 rpm for 30 min until uniform mixing. The mixture was then transferred to a Petri dish and degassed in a vacuum drying oven at 60–80 °C and an absolute pressure of −0.1 MPa for 4–6 h to remove residual solvent and bubbles. A crosslinkable viscous epoxy prepolymer was thus obtained, which was sealed and stored in a refrigerator at 4 °C for later use.

#### 2.2.4. Preparation of Profile Control Microsphere Suspension

The carrier fluid was freshly prepared before each experiment: HPAM powder was slowly added to the simulated injection water and stirred at 200 rpm for 2 h until fully dissolved to prepare an HPAM aqueous solution with a mass concentration of 3000 mg/L. The solution was left to stand for aging for 24 h before use.

The epoxy prepolymer was slowly added dropwise to the HPAM carrier fluid under high-speed stirring at 1000 rpm at a mass ratio of 1:10 (prepolymer to carrier fluid). After the dropwise addition, stirring was maintained for 10 min, followed by ultrasonic dispersion at 300 W for 2 min. Driven by the shear effect and interfacial tension of the aqueous phase, the epoxy prepolymer dispersed into uniform liquid microspheres in the aqueous phase, forming the high-temperature-resistant epoxy-based profile control microsphere system. This system is an oil-in-water suspension with liquid microspheres, and it was used for injection immediately after preparation to avoid premature crosslinking at the surface. After being injected into the core, it gradually cross-links and cures at formation temperature to achieve deep plugging.

#### 2.2.5. Curing Process

① First stage (pre-curing): The system was held at a constant temperature of 65 °C for 4–6 h, during which the amino groups of KH-550 completed the preliminary reaction with epoxy groups. Meanwhile, the oxazine rings of BPS-PH opened synchronously under the catalytic effect, rapidly generating phenolic hydroxyl catalytic sites.

② Second stage (deep curing): The system was further maintained at 65 °C for 12–16 h, during which the phenolic hydroxyl groups catalyzed the trimerization crosslinking of phthalonitrile groups. The full curing process was completed after a total constant-temperature holding of 16–22 h, forming a dense three-dimensional network structure.

**Figure 5:**
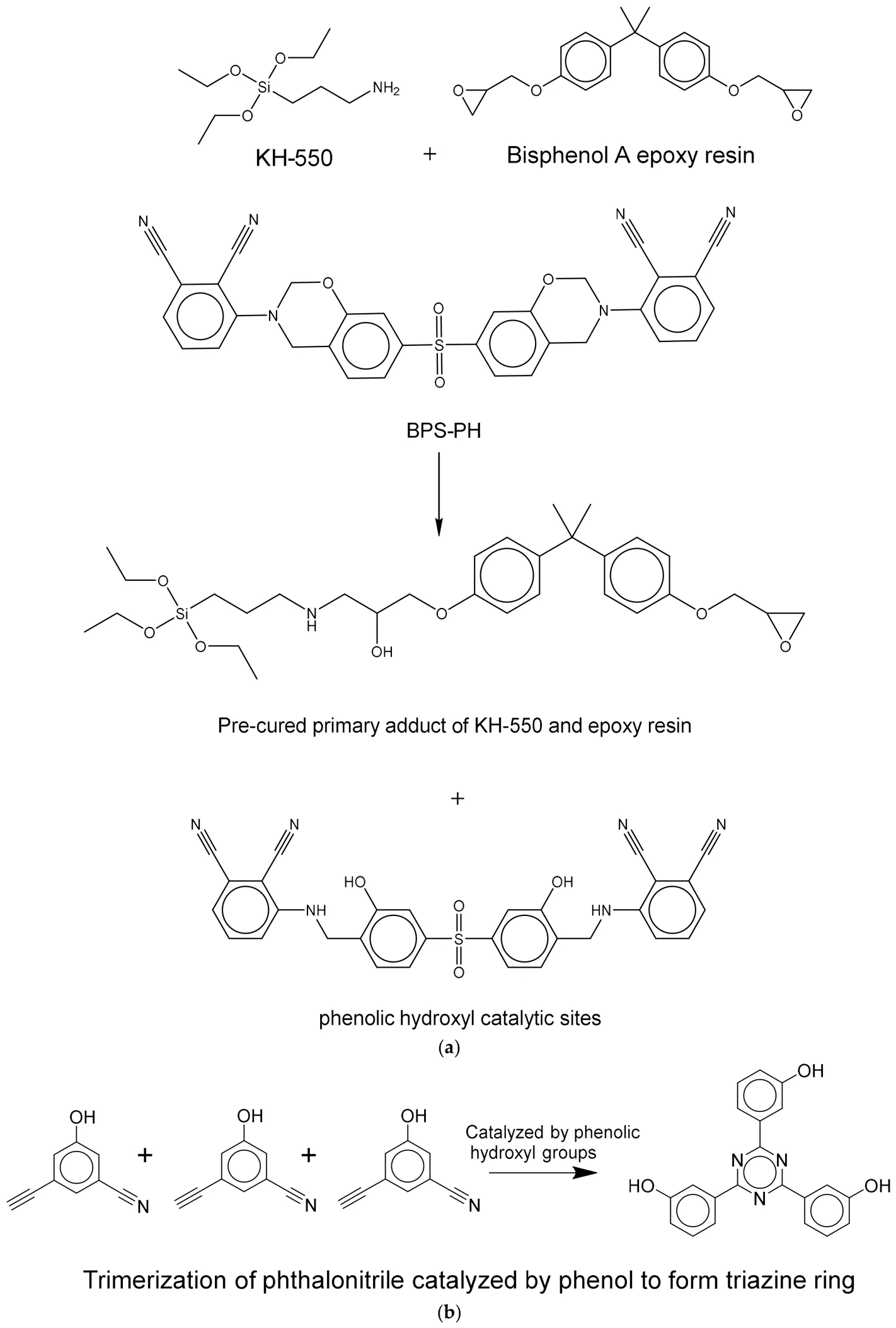
Schematic illustration of the two-stage curing process of the modified epoxy system. (**a**) Schematic of two parallel reactions during the first-stage pre-curing at 65 °C; (**b**) trimeric crosslinking.

The trimerization crosslinking mechanism of the phthalonitrile group during the two-stage curing procedure is illustrated in Figure 5.

### 2.3. Structural Characterization and Thermal Performance Tests

Fourier transform infrared spectroscopy (FT-IR) was performed using the KBr pellet method, with a spectral range of 4000–400 cm^−1^ and a resolution of 4 cm^−1^, to analyze the structural changes in functional groups. Proton nuclear magnetic resonance (^1^H NMR) tests were conducted with deuterated dimethyl sulfoxide (DMSO-d_6_) as the solvent at a frequency of 500 MHz to verify the formation of characteristic groups in the molecular structure. Thermogravimetric analysis (TGA) was employed to evaluate the thermal stability of the system. The tests were carried out under a nitrogen atmosphere with a flow rate of 50 mL/min, a heating rate of 10 °C/min, and a temperature range of 30–800 °C, with a sample mass of 5–10 mg. The mass loss at 350 °C was taken as the core evaluation index for formula optimization.

All characterization tests were repeated in triplicate for each sample. TGA mass loss data are presented as mean values, with the standard deviation of parallel samples controlled below 0.3%, ensuring the statistical reliability of thermal stability conclusions.

### 2.4. Displacement Performance Evaluation Experiments

All displacement experiments were performed in triplicate under identical conditions. Calculated parameters including resistance factor, residual resistance factor, plugging efficiency and oil recovery are expressed as mean values, with relative standard deviations of all results within 5%, guaranteeing good reproducibility of experimental data.

Sand-packed tube models were prepared by filling with quartz sand. Before experiments, the models were subjected to vacuum saturation with simulated formation water, and the pore volume, porosity and water-phase permeability were measured. The flow rates of all displacement experiments were expressed as equivalent water velocity. The specific experimental methods are as follows.

#### 2.4.1. Evaluation of Migration Performance

Water flooding was conducted at 3 mL/min at 65 °C until the pressure at each monitoring point stabilized, and the initial pressure was recorded. Then 1 pore volume (PV) of the profile control system suspension (with HPAM solution as the carrier fluid and a mass ratio of resin to carrier fluid of 1:10) was injected at the same flow rate. During injection, the pressure at each monitoring point and the produced fluid volume were recorded every 5 min. After injection, the system was allowed to cure at 65 °C for 24 h. After full curing, water flooding was resumed until the pressure stabilized, and the stable pressure was recorded. At the onset of subsequent water flooding, pressure build-up was required to match the pressure at the end of agent injection.

The pressure difference ratio *β* between adjacent monitoring points was calculated, and *β* < 4 was adopted as the evaluation criterion for deep migration capacity, with the formula shown as follows.

$$\beta_{1}=\frac{∆P_{in\sim 1}}{∆P_{1\sim 2}},\beta_{n}=\frac{∆P_{n-1\sim n}}{∆P_{n\sim out}}$$

Δ*P*_in~1_ = *P*_in_–*P*_1_, Δ*P*_1~2_ = *P*_1_–*P*_2_, …Δ*P_n_*_~out_ = *P_n_*–*P*_out_

where *P*_in_ is the pressure at the injection end, *P*_1_ is the pressure at monitoring point 1, …, *P*_out_ is the pressure at the production end, and “*n*” is the number of pressure monitoring points along the core length.

#### 2.4.2. Evaluation of Steam Flooding Plugging Performance

After completing the injection, curing and subsequent water flooding of the profile control system following the above procedures, the post-plugging permeability was measured. Then the steam generator (Jiangsu Haian Huada Petroleum Instrument Co., Ltd., Nantong, China) was connected, with a backpressure of 3 MPa set at the production end. 1 PV of saturated steam at 350 °C was injected at an equivalent water velocity of 10 mL/min. After steam flooding, the system was cooled to 65 °C and the water-phase permeability was remeasured. The plugging retention rate after steam scouring was calculated to evaluate the steam scouring resistance of the system.

Meanwhile, the resistance factor *F*_R_, residual resistance factor *F*_RR_ and plugging rate *η* were calculated, with the formulas shown as follows:

$$F_{R}\;=\;\frac{\Delta P_{p}}{\Delta P_{w}}$$

$$F_{\mathrm{RR}}=\frac{\Delta P_{f}}{\Delta P_{w}}$$

$$\eta =\frac{{K_{w}-K}_{f}}{K_{w}}\times 100\%$$

where Δ*P*_w_ is the water flooding pressure difference, Δ*P*_p_ is the agent injection pressure difference, and Δ*P*_f_ is the subsequent water flooding pressure difference, all in MPa; *K*_w_ and *K*_f_ represent the water-phase permeabilities of the sand-packed tube before and after plugging, respectively.

#### 2.4.3. Evaluation of Profile Control and Flooding Performance in Dual-Tube Parallel Model

A heterogeneous model was constructed by connecting high-permeability (approximately 10,000 × 10^−3^ μm^2^) and low-permeability (approximately 2000 × 10^−3^ μm^2^) sand-packed tubes in parallel. Oil flooding was conducted at 65 °C to displace formation water until no water was produced. After saturation with simulated oil, the model was aged for 12 h. Saturated steam at 300 °C was injected until the water cut of the produced fluid reached 90%, and the oil recovery of the steam flooding stage was recorded.

Subsequently, 0.3 pore volume (PV) of the profile control and flooding system (10% profile control agent carried by 3000 mg/L HPAM solution) was injected at 3 mL/min, followed by 0.2 PV of water flooding to drive the system to migrate deep into the formation. After 24 h of curing, steam flooding was resumed until the water cut reached 98%. The variations in pressure, water cut and oil recovery throughout the entire process were recorded.

## 3. Results and Discussion

### 3.1. Structural Characterization and Thermal Stability Formula Optimization

The structures of the epoxy resin before and after modification were characterized via Fourier transform infrared spectroscopy and proton nuclear magnetic resonance spectroscopy to verify the successful introduction of heat-resistant groups, including imide and triazine rings. On this basis, the single-factor variable method was adopted to investigate the effects of BPS-PH monomer, KH-550 curing agent, and modified nano-SiO_2_ dosage on the thermal stability of the system. The mass loss at 350 °C was taken as the core evaluation index for formula optimization, and the addition of all components was calculated based on the total mass of the chain-extended epoxy resin.

#### 3.1.1. Fourier Transform Infrared Spectroscopy Analysis

**Figure 6:**
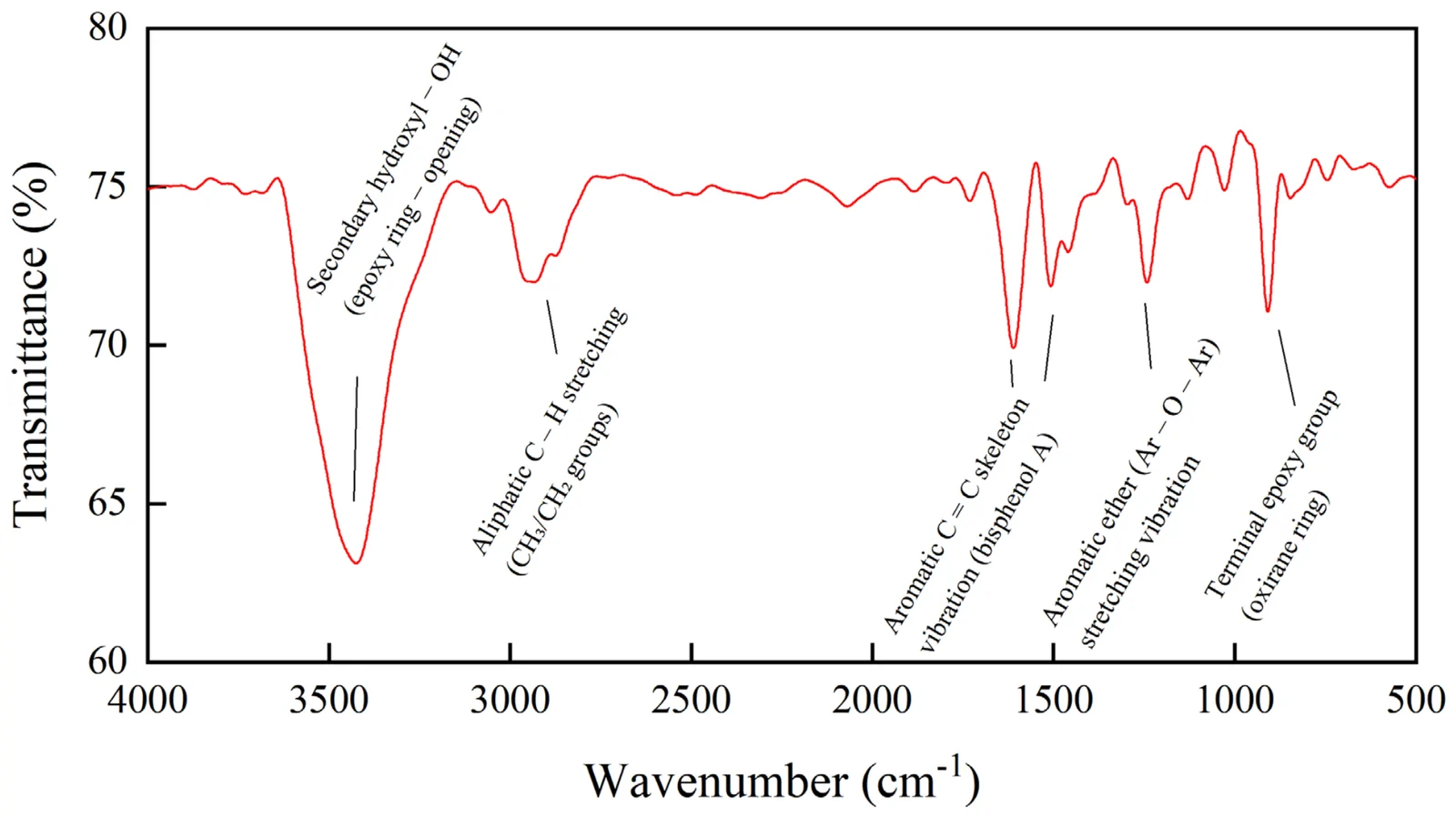
IR Spectrum of base epoxy prepolymer.

**Figure 7:**
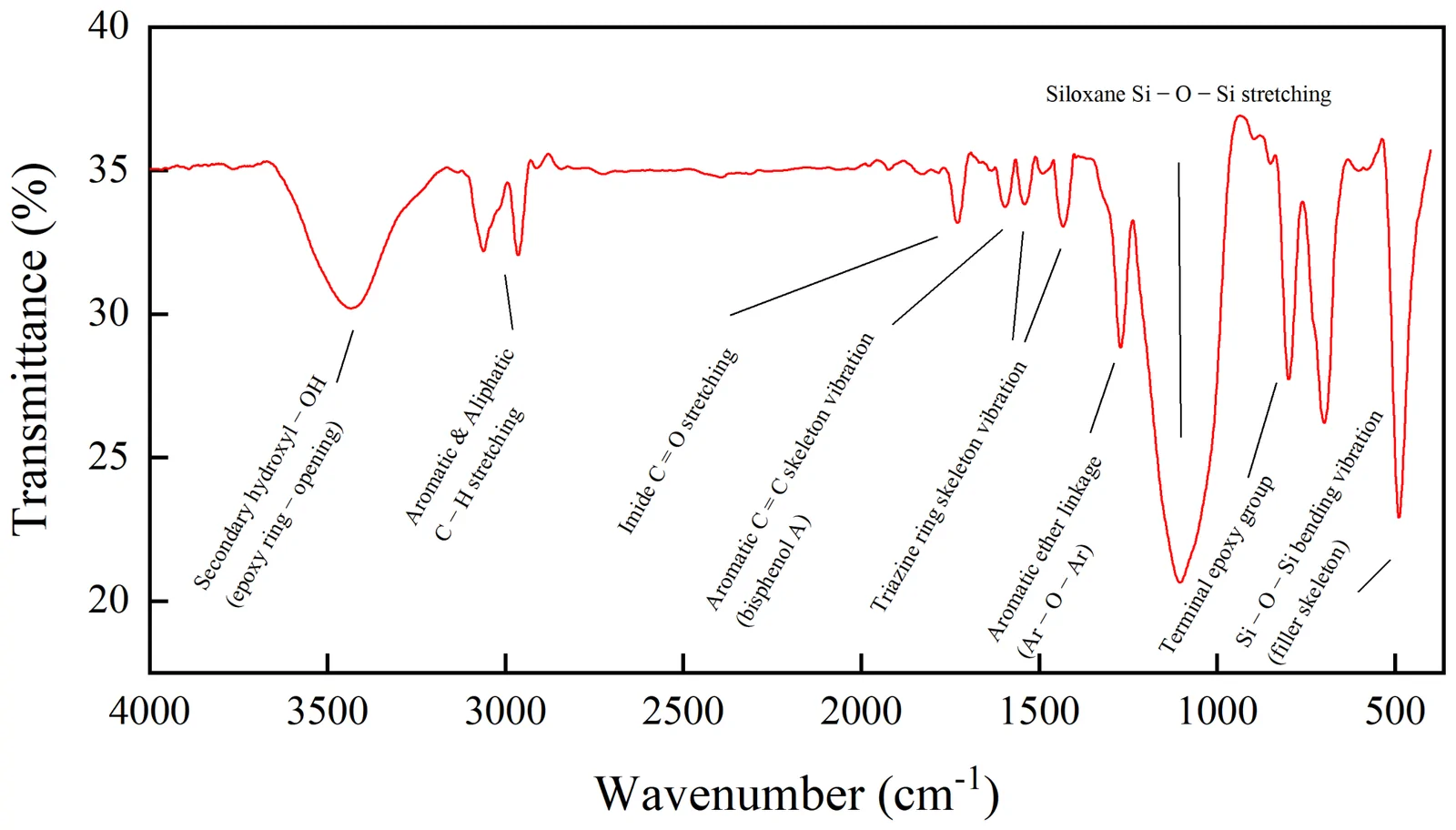
FT-IR spectrum of novel thermally stable epoxy resin.

The infrared spectra of the base epoxy prepolymer and the modified high-temperature resistant epoxy resin are shown in Figure 6 and Figure 7, respectively.

As shown in Figure 6, the base epoxy prepolymer exhibits a typical characteristic absorption peak of epoxy end groups at 910–830 cm^−1^, a stretching vibration peak of aromatic ether bonds at 1240 cm^−1^, a broad absorption peak of secondary hydroxyl groups around 3450 cm^−1^, and benzene ring skeleton vibration peaks at 1600 cm^−1^ and 1500 cm^−1^. These features are consistent with the standard structural characteristics of bisphenol A epoxy resin, indicating that no heat-resistant functional groups have been introduced into the system at this stage.

As shown in Figure 7, in addition to retaining the above basic characteristic peaks, the modified system presents a characteristic absorption band of nano-SiO_2_ in the range of 400–600 cm^−1^, a significantly enhanced absorption peak of Si-O-Si bonds at 1100 cm^−1^, skeleton vibration peaks of triazine rings at 1400 cm^−1^ and 1520 cm^−1^, as well as asymmetric and symmetric stretching vibration peaks of C=O from imide structures at 1780 cm^−1^ and 1720 cm^−1^. These changes in characteristic peaks confirm that the ring-opening of BPS-PH monomers successfully catalyzes the formation of triazine ring crosslinked structures, the imide rigid chain segments have been incorporated into the resin backbone, and the modified nano-SiO_2_ forms chemical bonding with the resin matrix via the coupling effect of KH-550. The heat-resistant modified structure is thus successfully constructed.

#### 3.1.2. Nuclear Magnetic Resonance Spectroscopy Analysis

**Figure 8:**
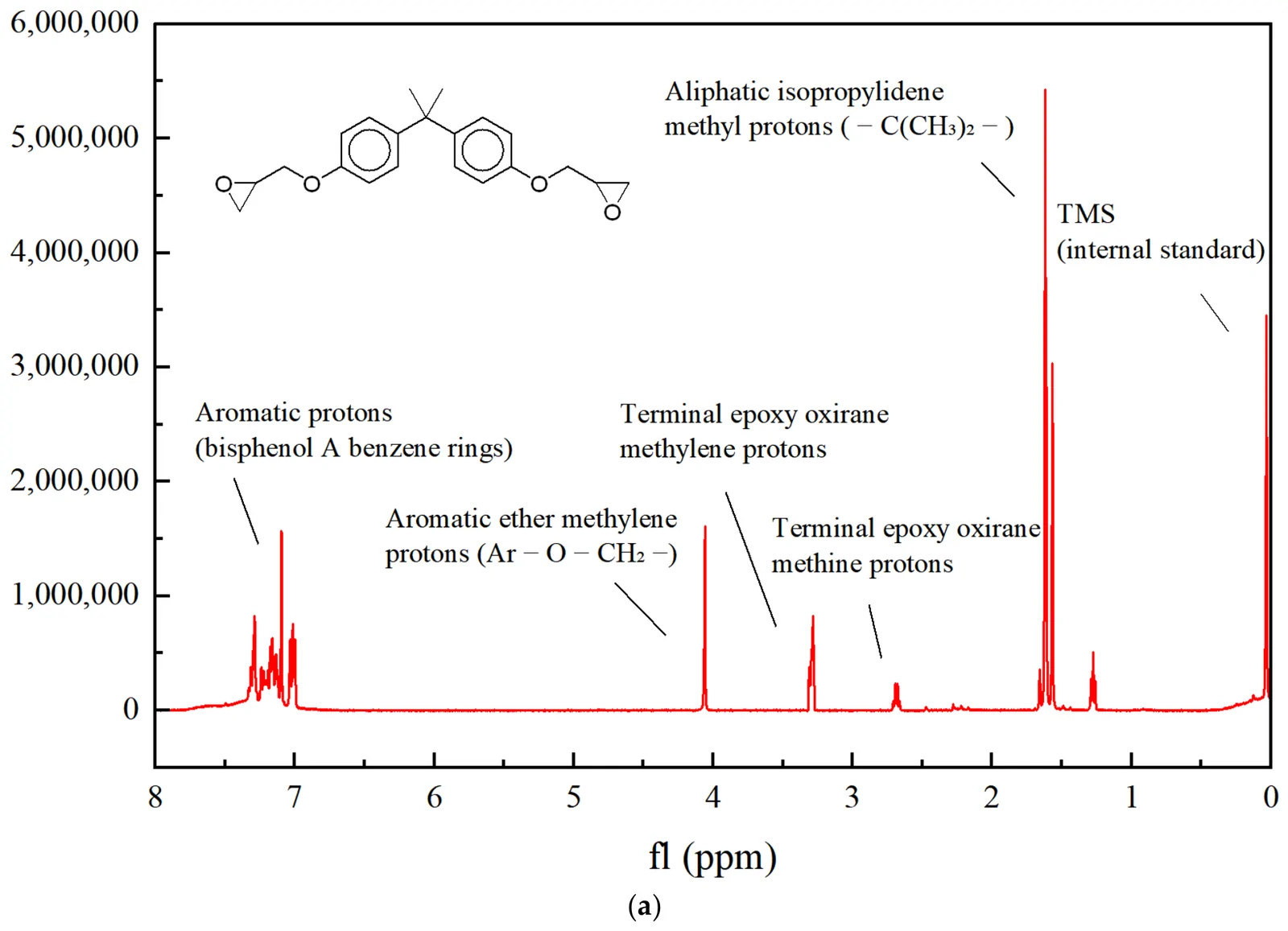

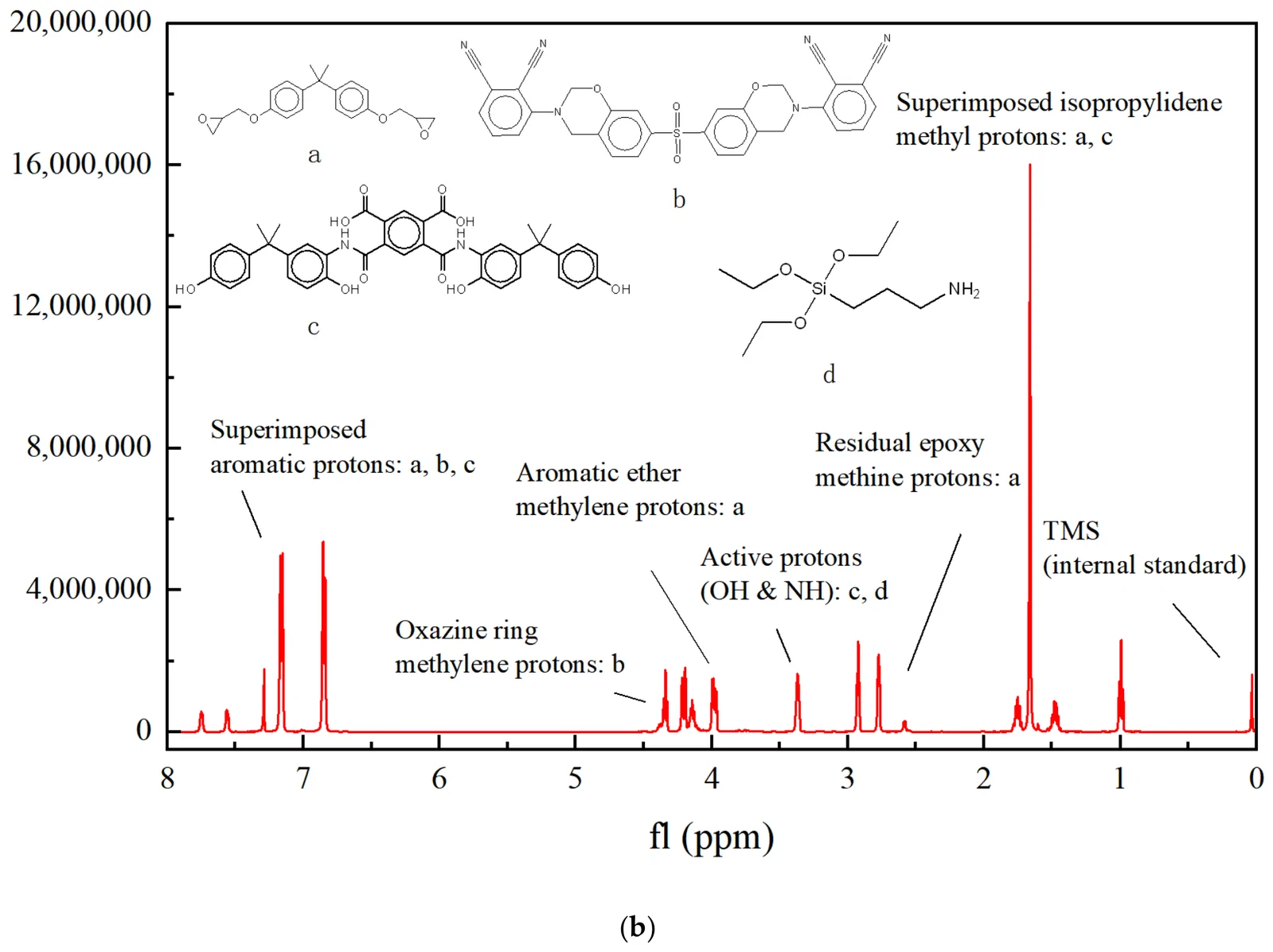
NMR test results. (**a**) Cured base epoxy resin; (**b**) cured thermally stable epoxy resin. Letters—a: Diepoxy monomer, b: BPS-PH, c: Polyamic-acid intermediate, d: KH-550.

The ^1^H NMR spectra of the base epoxy resin and the heat-resistant epoxy resin are presented in Figure 8.

In the spectrum of the base epoxy resin, the multiplet around δ = 7.0 ppm corresponds to aromatic protons on benzene rings, the doublet at δ = 2.5–2.8 ppm is assigned to methylene protons of epoxy groups, and characteristic proton peaks of aliphatic chain segments appear in the range of δ = 0.8–1.2 ppm. These features are fully consistent with the molecular structure of bisphenol A epoxy resin.

In the spectrum of the modified heat-resistant epoxy resin, characteristic proton peaks of triazine ring structures emerge at δ = 8.2–8.7 ppm. The intensity of proton peaks from amino and imide structures is significantly enhanced in the range of δ = 1.0–3.0 ppm, while the intensity of characteristic peaks of epoxy groups is markedly reduced. This result further verifies that the reaction processes of oxazine ring-opening, triazine ring crosslinking and imide chain segment introduction are successfully completed and the system achieves the expected molecular modification effect.

#### 3.1.3. Thermal Stability and Formula Optimization

On the basis of structural verification, the system formula was optimized via thermogravimetric analysis, and the effects of three core components on thermal stability were investigated separately.

**Figure 9:**
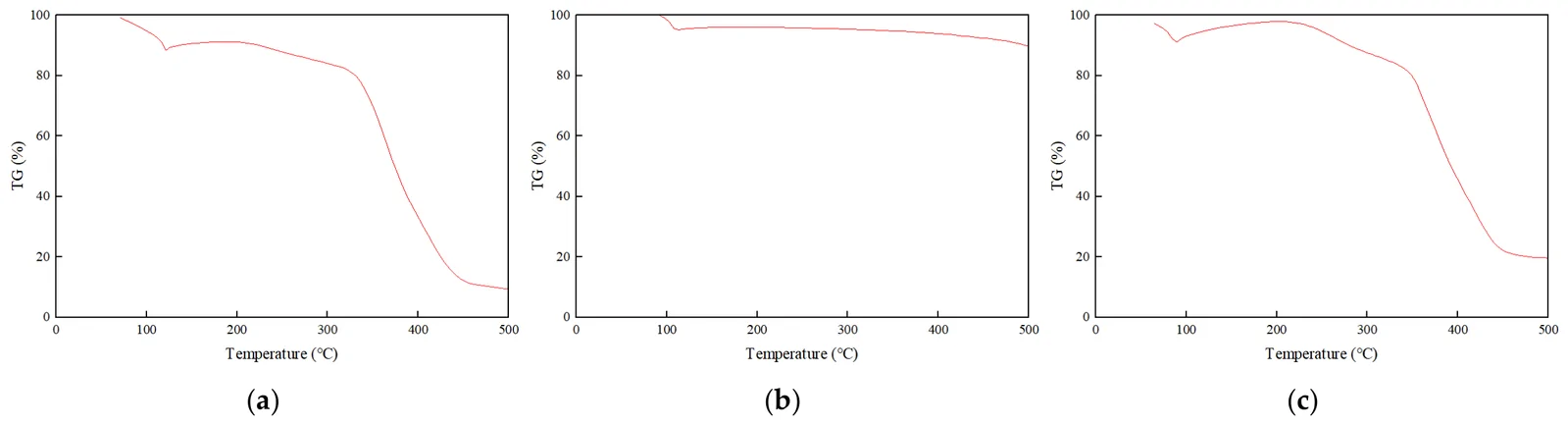
TGA results of epoxy resins with different BPS-PH contents. (**a**) 5 wt% BPS-PH; (**b**) 10 wt% BPS-PH; (**c**) 20 wt% BPS-PH.

The thermogravimetric analysis results of the heat-resistant epoxy resin with different BPS-PH contents are shown in Figure 9.

**Figure 10:**
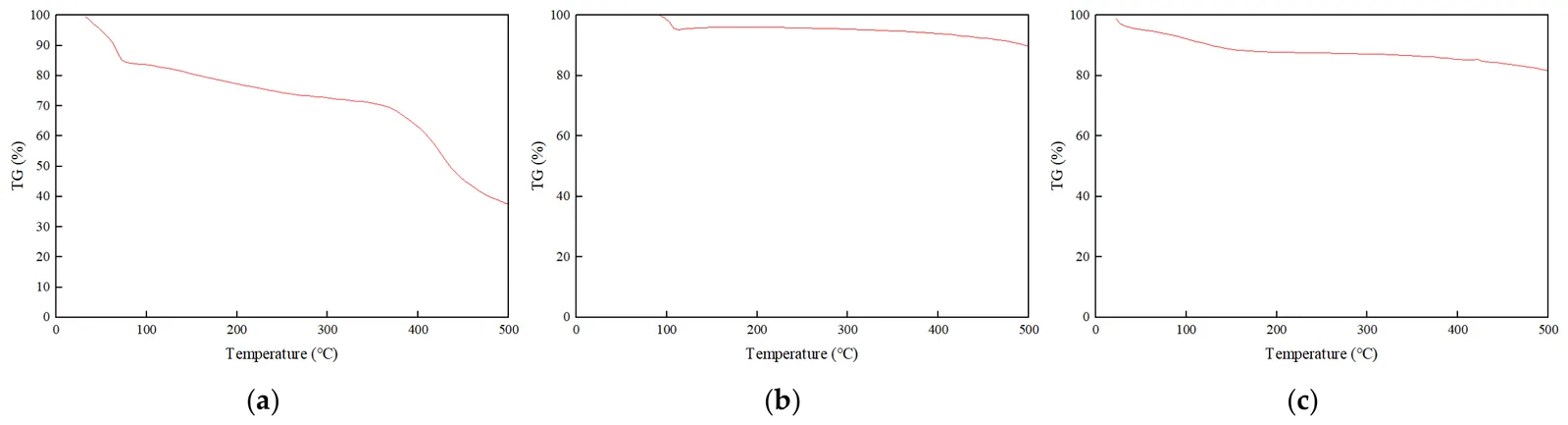
TGA curves of epoxy resins with varied KH-550 loadings. (**a**) 1 wt% KH-550; (**b**) 3 wt% KH-550; (**c**) 5 wt% KH-550.

The thermogravimetric analysis results of the heat-resistant epoxy resin with different KH-550 contents are shown in Figure 10.

**Figure 11:**
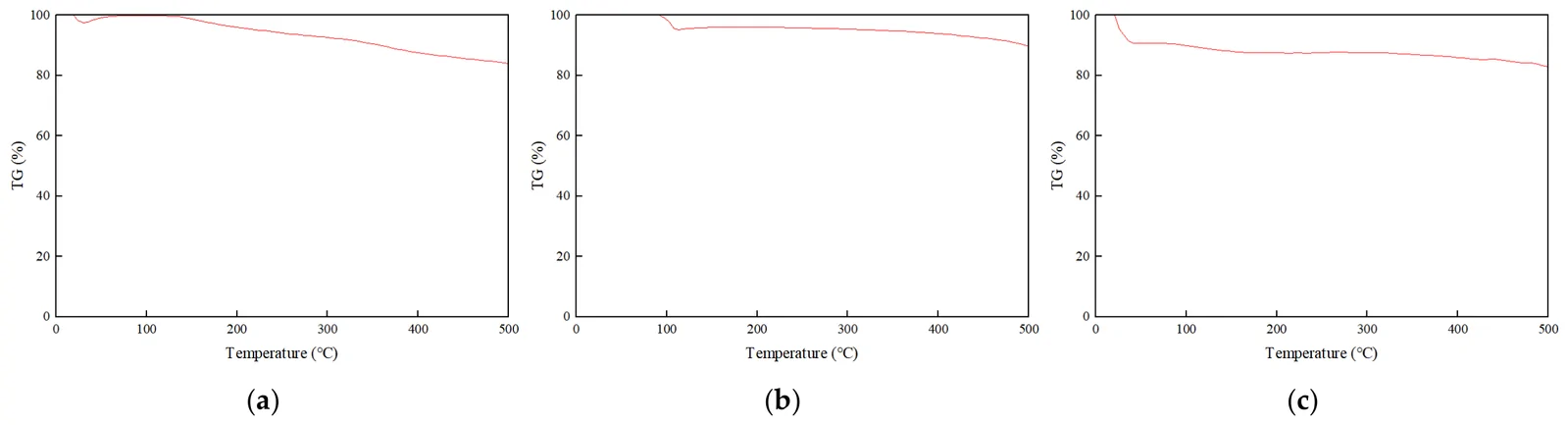
TGA results of epoxy resins with different nano-SiO_2_ contents. (**a**) 1 wt% nano-SiO_2_; (**b**) 2.5 wt% nano-SiO_2_; (**c**) 5 wt% nano-SiO_2_.

The thermogravimetric analysis results of the heat-resistant epoxy resin with different nano-SiO_2_ contents are shown in Figure 11.

##### Effect of BPS-PH Content

The thermogravimetric curves of the system with different BPS-PH contents are shown in Figure 9. With the KH-550 content fixed at 3% and nano-SiO_2_ content at 2.5%, when the BPS-PH content is 5%, the mass loss of the system at 350 °C exceeds 30%, indicating poor temperature resistance. When the content increases to 10%, the mass loss at 350 °C drops below 5%, achieving optimal thermal stability. When the content further rises to 20%, the mass loss at 350 °C rebounds to over 20%, leading to deteriorated temperature resistance.

The underlying mechanism is as follows: during the curing process, the oxazine rings of BPS-PH open to generate phenolic hydroxyl groups, which reduce the activation energy of the trimerization reaction of phthalonitrile groups, catalyze the formation of triazine ring crosslinked structures, and improve the crosslinking network density and thermal stability. Insufficient content leads to inadequate catalytic sites and imperfect crosslinking networks, resulting in limited heat resistance improvement. Excessive content causes an excessively fast curing reaction rate and concentrated internal heat release, which easily induces material embrittlement, micro-defects, and internal stress, and, in turn, deteriorates high-temperature structural stability. Therefore, the optimal BPS-PH dosage is 10%.

##### Effect of KH-550 Content

The thermogravimetric curves of the system with different KH-550 contents are shown in Figure 10. With the BPS-PH content fixed at 10% and nano-SiO_2_ content at 2.5%, when the KH-550 content is 1%, the mass loss of the system at 350 °C is approximately 25%; when the content increases to 3%, the mass loss decreases to about 5%, achieving optimal heat resistance; when the content further rises to 5%, the mass loss rebounds to around 13%.

KH-550 serves dual functions as both a curing agent and a coupling agent: amino groups undergo ring-opening crosslinking with epoxy groups to build the resin network, while siloxane groups form interfacial chemical bonding with nano-SiO_2_. Insufficient content results in sparse crosslinking nodes, insufficient compactness of the resin network, and inadequate interfacial bonding of nanofillers, leading to limited improvement in heat resistance. Excessive content tends to trigger self-condensation side reactions of siloxane groups, which disrupts the uniformity of the crosslinking network and reduces effective coupling sites, thereby decreasing thermal stability. Accordingly, the optimal KH-550 dosage is 3%.

##### Effect of Nano-SiO_2_ Content

The thermogravimetric curves of the system with different nano-SiO_2_ contents are shown in Figure 11. With the BPS-PH and KH-550 contents fixed at 10% and 3% respectively, when the nano-SiO_2_ content is 1%, the mass loss at 350 °C is approximately 10%, with an insignificant reinforcement effect; at 2.5% content, the mass loss drops to about 5%, achieving optimal performance; when the content rises to 5%, the mass loss rebounds to around 13%.

Nano-SiO_2_ extends the diffusion path of thermal decomposition products through the physical barrier effect, thus increasing the thermal decomposition activation energy. Meanwhile, surface-grafted amino groups participate in the curing reaction and increase crosslinking nodes. Insufficient content leads to a weak barrier effect and limited reinforcement. Excessive content causes nanoparticle agglomeration, which increases system viscosity and forms stress concentration points, thereby reducing the thermal stability and mechanical properties of the system. Hence, the optimal nano-SiO_2_ dosage is 2.5%.

In summary, the optimal formula of the system is determined as 10% BPS-PH, 3% KH-550, and 2.5% modified nano-SiO_2_. Under this formula, the average mass loss of three parallel tests at 350 °C is less than 5%, with a standard deviation below 0.3%, which meets the high-temperature working condition requirements above 300 °C in steam flooding. For subsequent performance evaluations, the epoxy prepolymer prepared with this optimal formula was used, and the liquid microsphere suspension was freshly prepared before each experiment.

### 3.2. Migration Performance in Porous Media and Its Influencing Factors

#### 3.2.1. Effect of Core Permeability on Migration Performance

Experiments were conducted in sand-packed tubes with different water-phase permeabilities, with the mass fraction of epoxy profile control microspheres fixed at 10%. The results of resistance factor, residual resistance factor and plugging rate are listed in Table 2, and the interval pressure differences and pressure difference ratios at the end of injection and the end of subsequent water flooding are presented in Table 3 and Table 4, respectively.

As can be seen from Table 2, Table 3 and Table 4, with the increase in water-phase permeability of the sand-packed tubes, the resistance factor and residual resistance factor of the system gradually decrease, and the interval pressure differences and pressure difference ratios along the flow path continuously decline. When the permeability is 5000 × 10^−3^ μm^2^, the pressure difference ratio between the first and last intervals in the injection stage reaches 4.723, slightly higher than the deep migration criterion. When the permeability increases to above 10,000 × 10^−3^ μm^2^, the pressure difference ratios of all intervals are below 4, and the ratios further decrease in the subsequent water flooding stage.

This pattern is directly related to the pore throat-droplet matching mechanism. The lower the permeability, the smaller the average pore throat size of the reservoir, which makes it easier for liquid microspheres to coalesce and retain in the pores, resulting in higher injection resistance and a larger proportion of front-end retention. With increasing permeability, the pore throat size expands, allowing liquid microspheres to pass through the pore structure smoothly via tensile deformation. This reduces the resistance to deep migration and leads to more uniform distribution along the flow path. Overall, the epoxy-based profile control system exhibits favorable deep migration capacity in the permeability range of 5000–15,000 × 10^−3^ μm^2^, which covers conventional high-permeability to ultra-high-permeability heavy oil reservoirs.

**Figure 12:**
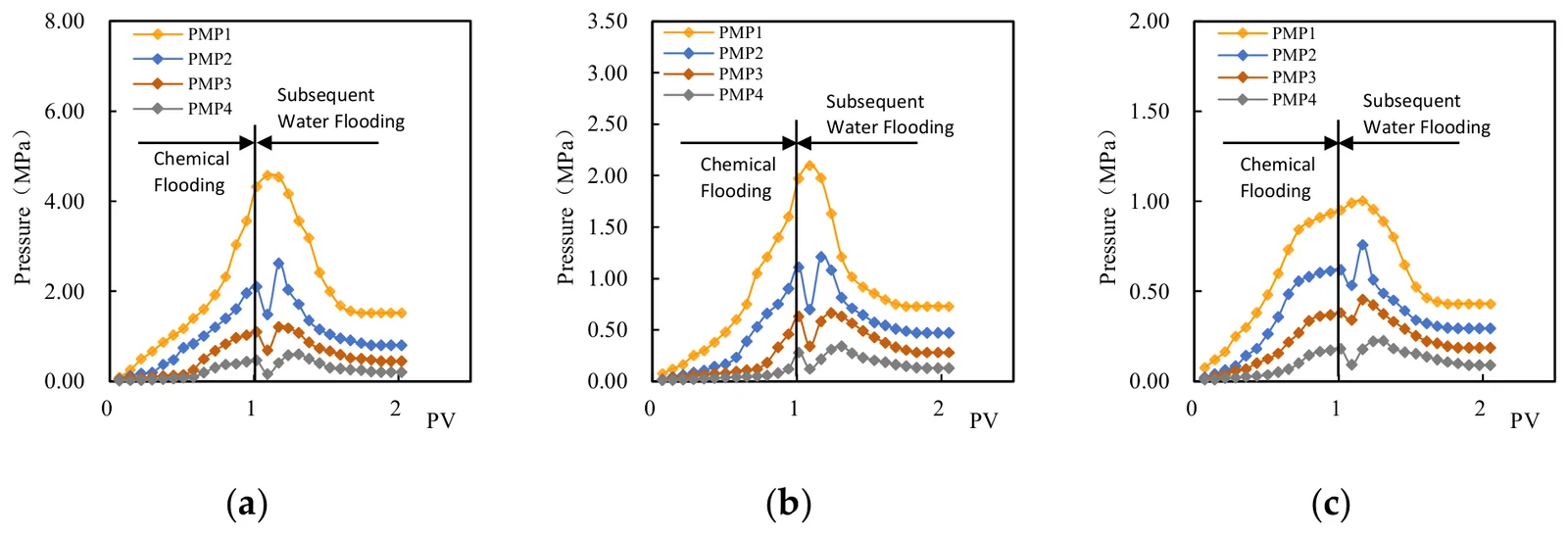
Relationships of core permeability and pressures at pressure measurement points vs. PV number (high-temperature resistant epoxy-based profile control microspheres). (**a**) *K*_w_ = 5000 × 10^−3^ μm^2^; (**b**) *K*_w_ = 10,000 × 10^−3^ μm^2^; (**c**) *K*_w_ = 15000 × 10^−3^ μm^2^.

The variation curves of pressure at each monitoring point with the number of injected pore volumes (PV) under different permeabilities are shown in Figure 12. During the injection stage, the pressure at each monitoring point rises gradually with increasing PV, and the pressure at the inlet monitoring point is consistently significantly higher than that at deep monitoring points. This is consistent with the distribution rule that droplets retain along the flow path, with greater retention at the front end. The lower the permeability, the higher the overall pressure level and the faster the pressure rise rate, which corresponds to the experimental result of a larger resistance factor. In the subsequent water flooding stage, the pressure all follows a trend of rapid decline followed by gradual stabilization. After stabilization, the pressure still maintains an along-flow distribution of high at the inlet and low at the outlet, indicating that the microspheres complete crosslinking and curing at formation temperature and form a stable plugging structure distributed along the flow path.

#### 3.2.2. Effect of Epoxy Resin Content on Migration Performance

With the water-phase permeability of the sand-packed tube fixed at 10,000 × 10^−3^ μm^2^, the mass fraction of the epoxy prepolymer was adjusted, and the experimental results are presented in Table 5, Table 6 and Table 7.

The results show that with the increase in epoxy system content, the resistance factor, residual resistance factor and plugging rate increase gradually, and the interval pressure differences and pressure difference ratios rise synchronously. At a content of 15%, the pressure difference ratio of the first interval during injection is still only 2.030, far below the deep migration criterion of 4.

The underlying reason is as follows: higher system concentration means more liquid microspheres per unit pore volume, greater coalescence and retention in pores, and improved plugging strength. Meanwhile, the injection resistance increases and the proportion of front-end retention rises, leading to a slight decline in deep migration capacity. Overall, the epoxy system can satisfy the requirements of both plugging strength and deep migration within the experimental concentration range.

Compared with 30 μm inorganic quartz sand particles, inorganic particles deliver a slightly higher resistance factor and initial plugging rate at the same content, but their pressure difference ratio is significantly higher than that of the epoxy system. During injection, the first-interval pressure difference ratio of inorganic particles reaches 4.755, and the ratio between the first and last intervals is as high as 9.511. Most particles bridge and retain at the core inlet section, and only a small fraction can penetrate into the deep zone.

The difference in migration performance between the two systems arises from their essential distinctions in morphology and deformation mechanism. Inorganic quartz sand is rigid solid particles without deformability. When the ratio of particle size to pore throat diameter is high, they readily bridge and plug at pore inlets and are difficult to migrate deeply. In contrast, the epoxy profile control microspheres are liquid droplets with excellent flow deformability. They can pass through pore throats smaller than their own particle size via stretching and deformation and are unlikely to form permanent bridging. Thus, they can effectively break through near-wellbore retention and migrate deep into the reservoir.

**Figure 13:**
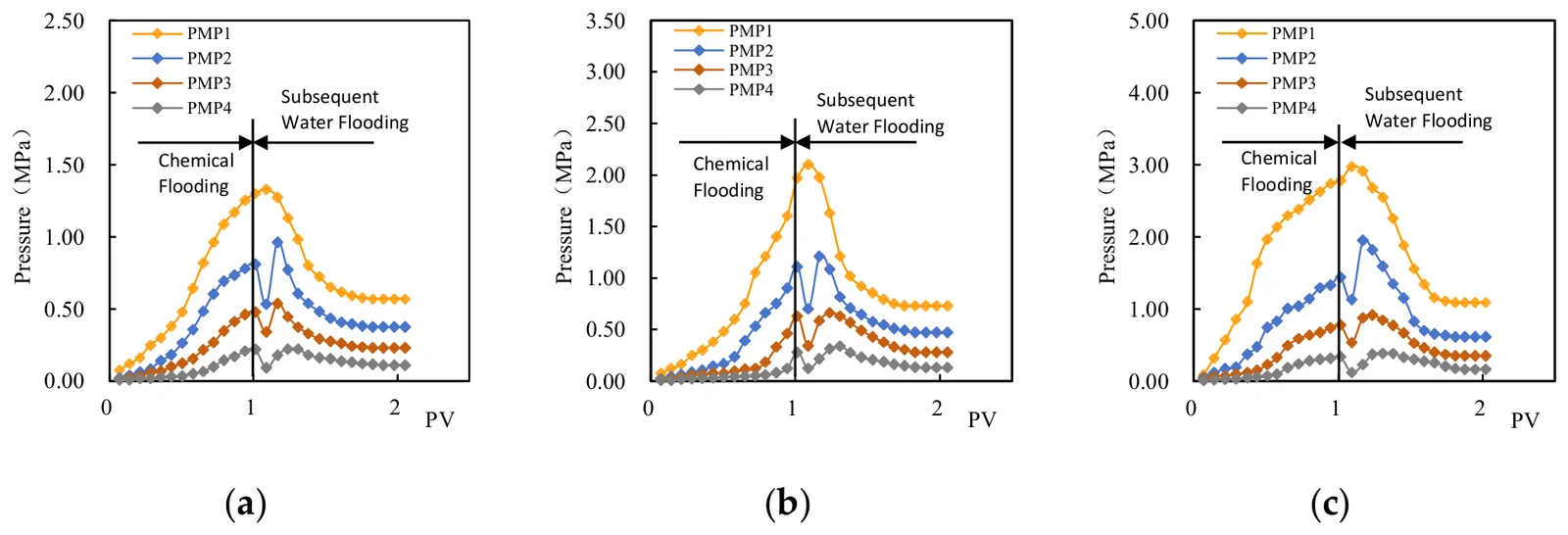
Relationships between epoxy resin content and pressures at pressure measurement points versus PV number. (**a**) 5 wt% epoxy resin; (**b**) 10 wt% epoxy resin; (**c**) 15 wt% epoxy resin.

**Figure 14:**
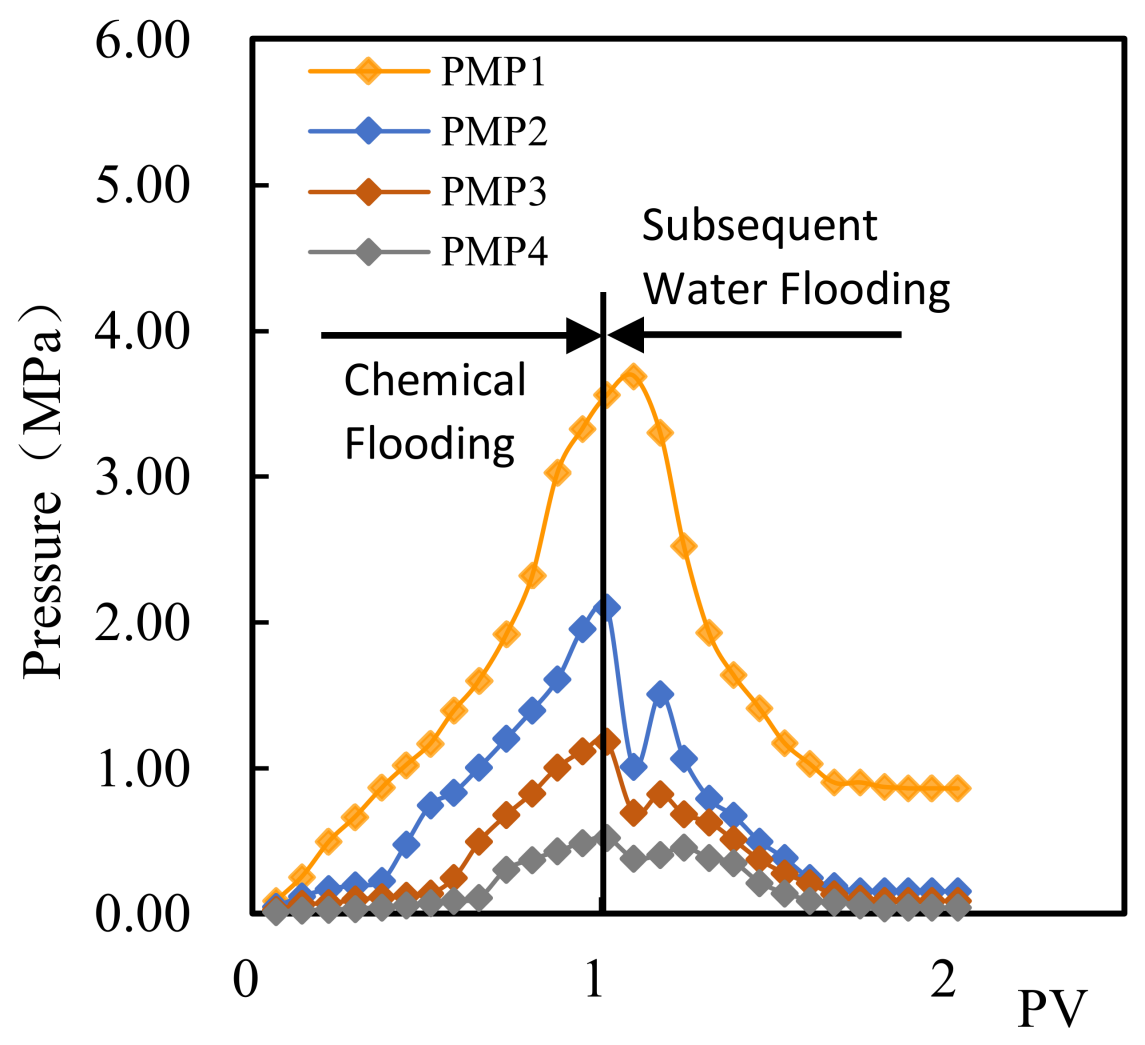
Relationship between pressures at pressure measurement points and PV number (10 wt% inorganic particles).

The variation curves of pressure at monitoring points with PV number under different system concentrations are shown in Figure 13 and Figure 14. During the injection stage, the injection pressure at each monitoring point rises synchronously with increasing epoxy system content, consistent with the trend of growing retention along the flow path. In the subsequent water flooding stage, the pressure all follows a trend of decreasing first, then stabilizing. The pressure at the inlet end is significantly higher than that at deep monitoring points, which is distinctly different from the distribution feature of inorganic particles—extremely high front-end pressure and extremely low deep pressure—further verifying the difference in migration capacity between the two systems.

### 3.3. Plugging Retention Performance Under High-Temperature Steam Scouring

Steam scouring experiments at 350 °C were conducted in sand-packed tubes with a water-phase permeability of approximately 10,000 × 10^−3^ μm^2^. After injection, the system was allowed to cure in situ at 65 °C for 24 h. The experimental results are presented in Table 8.

The results show that the plugging retention rate after steam flooding increases gradually with rising epoxy system concentration. After steam scouring, the plugging rate of each group decreases by 2–3%, with a slight reduction in plugging strength.

The decline in plugging rate is mainly attributed to two factors. First, at the high temperature of 350 °C, partial thermal degradation of HPAM in the carrier fluid weakens its adhesion and retention effect on the cured solids, reducing the retention stability of some small-sized cured particles in pores. Second, the mechanical scouring of high-velocity steam flow carries out partially crosslinked fine cured fragments with the fluid, slightly lowering the compactness of the plugging framework. Overall, however, the plugging rate of all groups remains above 95% after steam scouring, indicating that the three-dimensional crosslinked network formed by in situ curing of microspheres possesses excellent high-temperature steam scouring resistance and can meet the long-term plugging requirements of steam flooding.

Compared with inorganic quartz sand particles, the epoxy system delivers a slightly lower plugging rate after steam flooding, with a gap of less than 1 percentage point, and the two systems are at the same level of plugging strength. Combined with the aforementioned migration performance results, the epoxy system achieves significantly superior deep migration capacity while maintaining plugging strength comparable to inorganic particles. It realizes the synergistic effect of “deep migration + efficient high-temperature resistant plugging”, and addresses the technical limitation of conventional inorganic particles that only plug the near-wellbore zone rather than the deep reservoir.

### 3.4. Oil Displacement Effect and Action Mechanism of Deep Profile Control and Flooding in Heterogeneous Reservoirs

A dual-tube parallel heterogeneous model with high permeability (approximately 10,000 × 10^−3^ μm^2^) and low permeability (approximately 2000 × 10^−3^ μm^2^) was adopted to simulate the heterogeneous formation conditions of heavy oil reservoirs. The oil increment effect of the epoxy-based profile control and flooding system via deep profile control was evaluated and compared with the inorganic particle system. The experimental results are presented in Table 9 [21].

The oil recovery data show that after injection of the profile control and flooding systems, the oil recovery of both the high-permeability and low-permeability layers improves to varying degrees, but the performance of the two systems differs significantly. The inorganic particle system yields a slightly higher recovery increment in the high-permeability layer, but the increment in the low-permeability layer is only 12.44%, with an overall recovery increment of 8.44%. For the epoxy-based profile control and flooding system, the recovery increment in the low-permeability layer reaches 21.27%, and the overall recovery increment amounts to 12.05%, which is markedly superior to the inorganic particle system.

The difference in oil displacement performance between the two systems stems from the disparity in plugging positions and fluid diversion scope. Inorganic rigid particles have poor deformability and mainly remain in the near-wellbore zone of the high-permeability layer. They can only achieve fluid diversion near the wellbore, forcing steam into the near-wellbore area of the low-permeability layer. However, with continuous steam injection, the deep section of the high-permeability layer remains unplugged, and steam tends to bypass and re-enter high-permeability channels, resulting in limited improvement of deep swept volume and a low overall producing degree of the low-permeability layer.

In contrast, the epoxy-based profile control microspheres are liquid droplets that can migrate to the deep section of the high-permeability layer via flow deformation, then cure and plug in situ at formation temperature. This establishes fluid diversion along the full length of the well interval, continuously driving steam into the deep low-permeability layer and significantly expanding the steam-swept volume of the low-permeability layer. Accordingly, the recovery improvement of the low-permeability layer is more pronounced, and the overall oil increment effect is better. This result verifies that the system has excellent potential for deep profile control and steam channeling regulation in heterogeneous heavy oil steam flooding reservoirs.

## 4. Conclusions

To address the technical bottleneck of “difficulty in balancing high-temperature resistance and deep migration capacity” in steam channeling control for heavy oil steam flooding, this paper developed a high-temperature resistant epoxy-based profile control and flooding microsphere system. Through structural characterization, thermal performance optimization and sand-packed tube displacement experiments, the temperature resistance, migration performance, plugging performance and oil increment effect of the system were systematically evaluated. The main conclusions are drawn as follows:

(1) Using bisphenol A epoxy resin as the matrix, a high-temperature-resistant epoxy profile control and flooding system was successfully constructed via a modification route of imide chain extension, oxazine ring crosslinking and synergistic reinforcement of nano-SiO_2_. The optimal formula consists of 10% BPS-PH, 3% KH-550 and 2.5% modified nano-SiO_2_. Under this formula, the thermal mass loss rate of the system at 350 °C is less than 5%, which meets the high-temperature working condition requirements of over 300 °C in steam flooding. FT-IR and ^1^H NMR characterization results confirm that heat-resistant groups such as imide and triazine rings have been successfully introduced into the resin’s crosslinked network.
(2) The epoxy-based profile control and flooding system is composed of oil-in-water liquid microspheres. Its deep migration capacity is jointly affected by core water-phase permeability and system concentration: higher permeability and lower system concentration lead to smaller along-flow pressure difference ratios and stronger deep migration capacity. In the permeability range of 5000–15,000 × 10^−3^ μm^2^, the pressure difference ratios of all intervals of the system are generally below 4, and its migration performance is significantly superior to that of 30 μm rigid inorganic quartz sand particles. This advantage originates from the flow deformation property of liquid microspheres, which can pass through pore throats smaller than their own size via deformation and break through near-wellbore bridging and retention.
(3) After being injected into the core, the system can undergo in situ crosslinking and curing at formation temperature to form high-strength plugging. After scouring by high-temperature steam at 350 °C, the plugging rate of the system remains above 95%. Its plugging strength is at the same level as that of inorganic rigid particles, and it also exhibits excellent steam scouring resistance.
(4) Experiments on the heterogeneous dual-tube parallel model show that the system can achieve full-well-interval fluid diversion in high-permeability layers through deep migration and in situ curing, significantly expanding the steam swept volume of low-permeability layers. Compared with the inorganic particle system, the system delivers a notably higher oil recovery increment in low-permeability layers, with an overall recovery increment of up to 12.05%, showing better profile control and flooding performance. It can provide a new technical approach for deep steam channeling control in heavy oil steam flooding reservoirs.

## Figures and Tables

**Table 1:** Water quality analysis (mg/L).

Water-Type	Anion				Cation			Total Mineralisation
	K^+^ + Na^+^	Mg^2+^	Ca^2+^	Cl^−^	SO_4_^2−^	HCO_3_^−^	CO_3_^2−^	
Sewage	656	6	35	364	13	1027	50	2152

**Table 2:** Plugging performance parameters under different permeabilities.

Scheme	Profile Control and Displacement Agent	Permeability (10^−3^ μm^2^)	Resistance Factor (*F*_R_)	Residual Resistance Factor (*F*_RR_)	Plugging Efficiency (%)
1-1	Epoxy resin microspheres	5000	216.00	76.00	98.68
1-2		10,000	197.00	72.00	98.61
1-3		15,000	139.71	63.24	98.42
1-4	Inorganic particles	10,000	356.1	86.3	98.84

**Table 3:** Inter-zonal pressure difference δ*P* and pressure difference ratio *β* at the end of system injection under different permeabilities.

Scheme	System	Core Permeability *K*_w_ (10^−3^ μm^2^)	Pressure Difference δ*P* (MPa)				Pressure Difference Ratio *β*			
			δ*P*_1-2_	δ*P*_2-3_	δ*P*_3-4_	δ*P*_4-Outlet_	δ*P*_1-2_ /δ*P*_2-3_	δ*P*_2-3_ /δ*P*_3-4_	δ*P*_3-4_ /δ*P*_4-Outlet_	δ*P*_1-2_ /δ*P*_4-Outlet_
1-1	Epoxy resin microspheres	5000	2.220	1.000	0.630	0.470	2.220	1.587	1.340	4.723
1-2		10,000	0.860	0.480	0.350	0.280	1.792	1.371	1.250	3.071
1-3		15,000	0.330	0.240	0.200	0.180	1.375	1.200	1.111	1.833

**Table 4:** Pressure difference δ*P* and pressure difference ratio *β* at the end of subsequent water flooding under different permeabilities.

Scheme	System	Core Water Permeability *K*_w_ (10^−3^ μm^2^)	Pressure Difference δ*P* (MPa)				Pressure Difference Ratio *β*			
			δ*P*_1-2_	δ*P*_2-3_	δ*P*_3-4_	δ*P*_4-Outlet_	δ*P*_1-2_ /δ*P*_2-3_	δ*P*_2-3_ /δ*P*_3-4_	δ*P*_3-4_ /δ*P*_4-Outlet_	δ*P*_1-2_ /δ*P*_4-Outlet_
1-1	Epoxy resin microspheres	5000	0.720	0.350	0.250	0.200	2.057	1.400	1.250	3.600
1-2		10,000	0.260	0.190	0.150	0.130	1.368	1.267	1.154	2.000
1-3		15,000	0.136	0.108	0.096	0.090	1.259	1.125	1.067	1.511

**Table 5:** Plugging performance parameters at different contents.

Scheme	Type of Profile Control and Displacement Agent	Permeability (10^−3^ μm^2^)	Content (%)	Resistance Factor (*F*_R_)	Residual Resistance Factor (*F*_RR_)	Plugging Efficiency (%)
2-1	Epoxy resin microspheres	10,000	15	278.00	109.00	99.08
2-2		10,000	10	197.00	72.00	98.61
2-3		10,000	5	130.00	57.00	98.25
2-4	Inorganic particles	10,000	10	356.1	86.3	98.84

**Table 6:** Inter-zonal pressure difference δ*P* and pressure difference ratio *β* at the end of system injection at different contents.

Scheme	System	Content (%)	Pressure Difference δ*P* (MPa)				Pressure Difference Ratio *β*			
			δ*P*_1-2_	δ*P*_2-3_	δ*P*_3-4_	δ*P*_4-Outlet_	δ*P*_1-2_ /δ*P*_2-3_	δ*P*_2-3_ /δ*P*_3-4_	δ*P*_3-4_ /δ*P*_4-Outlet_	δ*P*_1-2_ /δ*P*_4-Outlet_
2-1	Epoxy resin microspheres	15	1.340	0.660	0.440	0.340	2.030	1.500	1.294	3.941
2-2		10	0.860	0.480	0.350	0.280	1.792	1.371	1.250	3.071
2-3		5	0.490	0.330	0.260	0.220	1.485	1.269	1.182	2.227
2-4	Inorganic particles	10	1.807	0.380	0.210	0.190	4.755	1.810	1.105	9.511

**Table 7:** Pressure difference δ*P* and pressure difference ratio *β* at the end of subsequent water flooding at different contents.

Scheme	System	Content (%)	Pressure Difference δ*P* (MPa)				Pressure Difference Ratio *β*			
			δ*P*_1-2_	δ*P*_2-3_	δ*P*_3-4_	δ*P*_4-Outlet_	δ*P*_1-2_ /δ*P*_2-3_	δ*P*_2-3_ /δ*P*_3-4_	δ*P*_3-4_ /δ*P*_4-Outlet_	δ*P*_1-2_ /δ*P*_4-Outlet_
2-1	Epoxy resin microspheres	15	0.480	0.258	0.192	0.160	1.860	1.344	1.200	3.000
2-2		10	0.260	0.190	0.150	0.130	1.368	1.267	1.154	2.000
2-3		5	0.193	0.146	0.121	0.110	1.322	1.207	1.100	1.755
2-4	Inorganic particles	10	0.522	0.136	0.110	0.108	3.838	1.236	1.019	4.833

**Table 8:** Variation in plugging efficiency before and after steam flooding displacement.

Scheme	Type of Profile Control and Displacement Agent	Concentration of Profile Control System (%)	Initial Permeability (10^−3^ μm^2^)	Permeability After Plugging (10^−3^ μm^2^)	Plugging Efficiency (%)	Permeability After Steam Flooding (10^−3^ μm^2^)	Plugging Efficiency (%)
3-1	Epoxy resin microspheres	5	10,589	203	98.08	504	95.24
3-2		10	10,251	143	98.61	425	95.85
3-3		15	10,056	126	98.75	356	96.46
3-4	Inorganic particles	10	10,165	105	98.97	321	96.84

**Table 9:** Steam flooding recovery efficiency in heterogeneous models.

Scheme	Type of Profile Control and Displacement Agent	Dual-Tube Model	Permeability (10^−3^ μm^2^)	Oil Saturation (%)	Oil Recovery (%)		
					Steam Flooding	After Profile Control Injection	Increment
4-1	Polymer carrying epoxy resin microspheres	High-perm	10,154	80.24	69.56	75.5	5.94
		Low-perm	1985	76.54	28.23	49.5	21.27
		Overall	6069.5	78.95	51.45	63.5	12.05
4-2	Polymer carrying inorganic particles	High-perm	10,375	80.55	69.46	76.1	6.64
		Low-perm	2018	76.41	27.86	40.3	12.44
		Overall	6196.5	78.83	50.76	59.2	8.44

## Data Availability

The raw data supporting the conclusions of this article will be made available by the authors on request.

## References

[1] Xu C., Liu L., Yang Y., Kang Y., You Z. (2024). An Innovative Fracture Plugging Evaluation Method for Drill-in Fluid Loss Control and Formation Damage Prevention in Deep Fractured Tight Reservoirs. Fuel.

[2] Zhang N., Yin D., Bo W., Wang Y., Sun Y., Miao X., Cao G. (2026). Micromechanical Interaction of Inorganic Solid Particles with Astratigraphic Rocks and Their Transport Mechanism within Porous Media. Geosyst. Eng..

[3] Guo H., Xue C., Wang D. (2026). Study on a Polymer Gel System for Deep Profile Control in High-Temperature and High-Salinity Reservoirs. Processes.

[4] Guo H., Ge J., Li L., Liu M., Wang W. (2024). Development, Evaluation and Stability Mechanism of High-Strength Gels in High-Temperature and High-Salinity Reservoirs. J. Mol. Liq..

[5] Joshi D., Ramesh D.N., Prakash S., Saw R.K., Maurya N.K., Rathi K.B., Mitra S., Sinha O.P., Bikkina P.K., Mandal A. (2025). Formulation and Characterisation of Polymer and Nanoparticle-Stabilized Anionic Surfactant Foam for Application in Enhanced Oil Recovery. Surf. Interfaces.

[6] Li Y., Li J., Zhang W., Lin Q., Chen L., Weng Z., Xiao Y. (2024). Rigid and Crosslinkable Polyimide Curing Epoxy Resin with Enhanced Comprehensive Performances. Polymer.

[7] Liu K., Huang Z.H.G., Yin D.Y., Wang X.B. (2023). Pressure Prediction Model for Deep Profile Control of Expanded Granular/Viscoelastic Movable Gel Formulation in Low Permeability Reservoir. J. Dispers. Sci. Technol..

[8] Liu Y., Dai C., Wang K., Zou C., Gao M., Fang Y., Zhao M., Wu Y., You Q. (2017). Study on a Novel Cross-Linked Polymer Gel Strengthened with Silica Nanoparticles. Energy Fuels.

[9] Zeng H., Bi M., Tong Z., Du Y., Chen X., Jiang T. (2026). KH550, KH560, and KH570 Modified SiO_2_ Enhance the Moisture Resistance, Thermal Properties and Electrical Insulation Properties of Epoxy Resin: Based on Experiments and Molecular Dynamics Simulations. J. Non-Cryst. Solids.

[10] Jiang H., Yang H., Zhang X., Kang W., Wang R., Li H., Zhang S., Chen X., Peng L., Shi H. (2025). Advances of Polymer Microsphere and Its Application in Porous Media for Enhanced Oil Recovery. Adv. Colloid Interface Sci..

[11] Li J., Niu L., Lu X. (2019). Migration Characteristics and Deep Profile Control Mechanism of Polymer Microspheres in Porous Media. Energy Sci. Eng..

[12] Li Y., Zhou M., Huang J., Chen X., Luo H. (2024). Preparation of Polymer Microspheres as Oil Displacement Agent for Extra-High Temperature and Extra-High Salinity Reservoirs. Colloids Surf. A Physicochem. Eng. Asp..

[13] Lobanova M.S., Aleshkevich V.V., Yablokova M.Y., Morozov O.S., Babkin A.V., Kepman A.V., Avdeev V.V., Bulgakov B.A. (2023). Kinetics of the Oxidative Aging of Phthalonitrile Resins and Their Effects on the Mechanical Properties of Thermosets. Thermochim. Acta.

[14] Pu W., Zhao S., Wang S., Wei B., Yuan C., Li Y. (2018). Investigation into the Migration of Polymer Microspheres (PMs) in Porous Media: Implications for Profile Control and Oil Displacement. Colloids Surf. A Physicochem. Eng. Asp..

[15] Zhang S., Lu Y., Wang Y., Ren D., Ye J., Huang Y., Han R. (2025). Catalytic Polymerization of Mono-Benzoxazine Functionalized Phthalonitrile Resin by Lewis Acids: Mechanism and Properties. J. Appl. Polym. Sci..

[16] Gao C., Xiong R., Guo J., Kiyingi W., Song H., Wang L., Zhang W., Chen X. (2025). A Review of Chemical Viscosity Reducers for Heavy Oil: Advances and Application Strategies. Fuel Process. Technol..

[17] Huang J., Chen H., Zhang G., Fan X., Liu J. (2022). The Effect of Silane Coupling Agent on the Texture and Properties of In Situ Synthesized PI/SiO_2_ Nanocomposite Film. Nanomaterials.

[18] Shen J., Yang H., Liu D., Kang W., Jiang H., Hao J., Wang H., Lv Z., Turtabayev S. (2023). Effect of Inorganic Particles on the Rheological Properties of Nano-SiO_2_ Grafted Modified Polymers. Phys. Fluids.

[19] Waniek T., Omar H., Szymoniak P., Silbernagl D., Sturm H. (2024). The Influence of Water Released from Particles in Epoxy-Based Nanocomposites. J. Appl. Polym. Sci..

[20] Wu S., Peng C., Wua Z. (2024). Promotion on the Thermal and Mechanical Behaviors of Epoxy Resin Using Phthalonitrile and Functionalized-SiO2. Polym. Adv. Technol..

[21] Borovina A., Hincapie R.E., Clemens T., Hoffmann E., Wegner J. (2022). Selecting EOR Polymers through Combined Approaches—A Case for Flooding in a Heterogenous Reservoir. Polymers.

